# A sticky end for gastrointestinal helminths; the role of the mucus barrier

**DOI:** 10.1111/pim.12517

**Published:** 2018-03-04

**Authors:** C. Sharpe, D. J. Thornton, R. K. Grencis

**Affiliations:** ^1^ Manchester Immunology Group Wellcome Trust Centre for Cell‐Matrix Research Faculty of Biology, Medicine and Health Manchester Academic Health Science Centre University of Manchester Manchester UK

**Keywords:** goblet cell, innate immunity, mucosal immunity, *Nippostrongylus brasiliensis*, *Trichinella spiralis*, *Trichuris* spp

## Abstract

Gastrointestinal (GI) nematodes are a group of successful multicellular parasites that have evolved to coexist within the intestinal niche of multiple species. It is estimated that over 10% of the world's population are chronically infected by GI nematodes, making this group of parasitic nematodes a major burden to global health. Despite the large number of affected individuals, there are few effective treatments to eradicate these infections. Research into GI nematode infections has primarily focused on defining the immunological and pathological consequences on host protection. One important but neglected aspect of host protection is mucus, and the concept that mucus is just a simple barrier is no longer tenable. In fact, mucus is a highly regulated and dynamic‐secreted matrix, underpinned by a physical hydrated network of highly glycosylated mucins, which is increasingly recognized to have a key protective role against GI nematode infections. Unravelling the complex interplay between mucins, the underlying epithelium and immune cells during infection are a major challenge and are required to fully define the protective role of the mucus barrier. This review summarizes the current state of knowledge on mucins and the mucus barrier during GI nematode infections, with particular focus on murine models of infection.

## 1 INTRODUCTION

Intestinal nematodes are among the most common and widely distributed animal parasites of humans, estimated to infect over 2.5 billion of the world's population, the majority of infections occurring in children.^1^, ^2^ Among the most prevalent intestinal worms are the hookworm (*Ancylostoma duodenale* and *Necator americanus*), roundworm (*Ascaris lumbricoides*) and whipworm (*Trichuris trichiura*)*,* which are typically found endemic in developing and tropical countries. These infections are normally transmitted by soil and are chronic in nature, which is in part due to endemic regions often lacking intervention that can curb transmission (ie medicinal care, diagnosis tools, effective sanitation, protocols to prevent reinfection and efficient treatment plans).^3^ Globally, these infections are accountable for causing severe morbidity to over 300 million individuals.^4^ Clinical manifestations of infections include malnutrition, cognitive dysfunction, vitamin deficiencies and growth retardation,^1^, ^4^ which all severely impair the quality of life of affected individuals. Despite their prevalence, this group of parasitic infections is considered as “minor” and often neglected in clinical treatment.

Current research is focused on defining host‐protective responses that lead to parasite expulsion, which are exceedingly difficult to elucidate within infected human populations. However, studies using various well‐established laboratory models of GI nematode infections have greatly contributed to our knowledge in understating how the host coordinates immune responses associated with resistance. Perhaps, the most commonly used murine models of helminth infections include *Trichuris muris*,* Trichinella spiralis*,* Nippostronglylus brasiliensis* and *Heligmosomoides polygyrus,* and a summary of each parasites’ life cycle during infection is shown in Table 1.

**Table 1:** Commonly used murine gastrointestinal (GI) colonizing nematodes, describing the niche and life cycle of parasites

Murine GI nematode	Type of parasite	Life cycle	GI niche
*Trichuris muris*	Whipworm	After ingestion of embryonated eggs, they hatch and invade the epithelial layer of the caecum and proximal colon, undergoing 4 moults before becoming adults.	Caecum
*Trichinella spiralis*	Roundworm	Infection occurs via ingestion of L1 larvae found within the muscle of a previously infected host. L1 larvae invade epithelial cells of the small intestine where they rapidly moult to adulthood.	Small intestine
*Nippostrongylus brasiliensis*	Hookworm	L3 larvae penetrate the skin, pass through the vasculature to the airways and crawl up the bronchi to be swallowed into the GI tract where they inhabit the small intestine.	Small intestine
*Heligmosomoides polygyrus*	Roundworm/hookworm	Free‐living L3 larvae are ingested and penetrate the submucosa of the small intestine; they moult and then reemerge into the intestinal lumen of the small intestine remaining in the villi.	Small intestine

Typically, most laboratory models of intestinal helminth infections can elicit a strong CD4^+^ Th2‐mediated immune response. Immune characteristics associated with a Th2 mediated environment are the secretion of type 2 signature cytokines (IL‐4, IL‐13, IL‐9 and IL‐5), activation of Th2 cells, antibody class switching to IgG1 (in mice) and IgE, and induction of alternatively activated macrophages, eosinophils, basophils and mast cells.^5^ This response is often referred to as an “allergic” immune response and is associated with goblet cell hyperplasia. As goblet cells are the major source of mucins (the major macromolecule of the intestinal mucus barrier), the expansion of this cell type can lead to the increased secretion of mucins which can consequently lead to alterations in the protective properties of the mucus barrier. The altered barrier can directly or indirectly affect parasite establishment within the GI niche, thus impeding the ability of the parasite to productively interact with the host and to thrive and survive. The involvement of mucus as a protective barrier during GI nematode infection was initially identified in the early 1980s, whereby the “mucus‐trap” hypothesis was coined.^6^, ^7^, ^8^ It was demonstrated that during *T. spiralis* and *N. brasiliensis* infection, the parasites were surrounded by mucus prior to their expulsion, indicating a role for mucus to physically separate and prevent the establishment of parasites within their niche. This observation suggested a direct role for the mucus barrier as an effector mechanism to protect the host and aid parasite expulsion. Indeed, subsequent characterization of animal models for GI helminth infections and the development of protocols to assess mucosal barrier properties have allowed the development of robust systems to directly investigate aspects of mucus barrier function and properties in vivo. These studies have demonstrated that mucins and mucus‐associated proteins hold key roles in altering the intestinal niche to enhance parasite expulsion, thus contributing to immune‐mediated host protection.^9^, ^10^, ^11^ Further insight into the precise functional role(s) that mucins and mucus‐associated proteins play within the mucus barrier may uncover potential avenues for novel therapeutic targets to eradicate this group of important neglected tropical diseases.

In this short review, we discuss the nature and formation of the intestinal mucus barrier and its mucin components during homeostasis. We will provide details on how mucins form mucus and describe the complexities of mucin synthesis, structure and function. Furthermore, we will elaborate how the immune system controls mucin production and properties to produce a mucus barrier with effective host‐protective function to combat GI nematode infections. Together this will highlight that mucus is not just a passive physical barrier but is a highly regulated and dynamic defence mechanism, and an important part of a coordinated immune‐driven host response against GI nematode infections.

## 2 THE INTESTINAL MUCUS BARRIER

**Figure 1:**
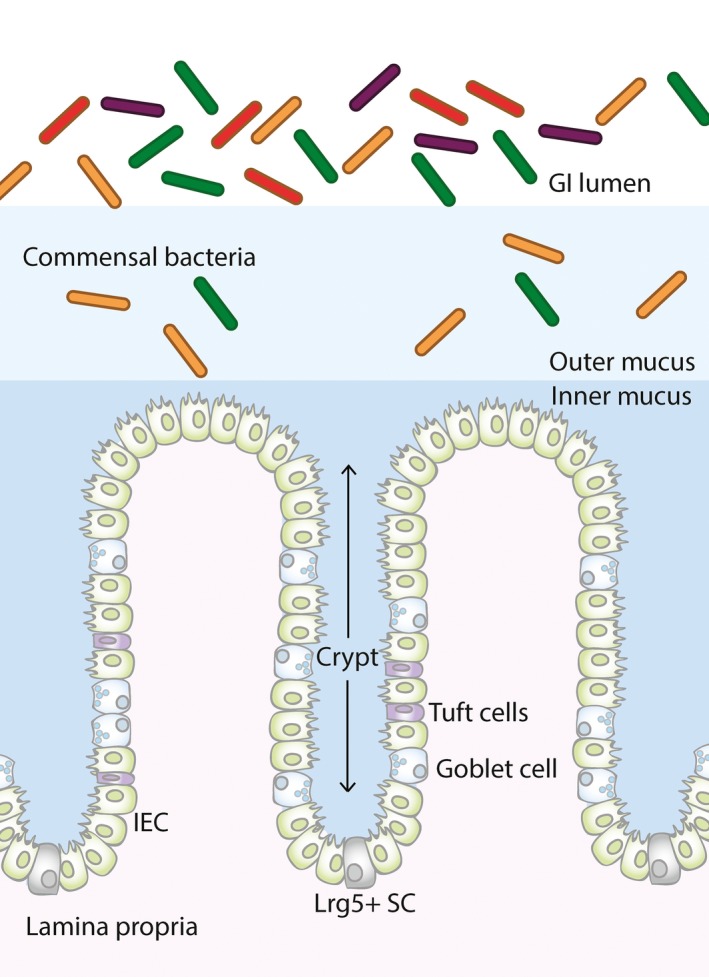
Illustration of the anatomy of the large intestine. The structural organization of the large intestine is shown in the illustration above. Lrg5^+^ stem cells (SC) are found at the base of the intestinal crypts and differentiate into mature lineages of surface epithelium cells, including intestinal epithelium cells (IEC), goblet cells and tuft cells. The large intestinal mucus barrier has 2 distinct structures: the loose outer mucus layer and the tightly adherent inner mucus layer. The outer mucus layer is colonized by commensal bacteria

The mucosa of the intestine is made up of a monolayer of cells arranged in multiple crypts that physically separates the external environment and subepithelium. The apical surface of the intestinal mucosal cells is protected by a carbohydrate‐rich barrier comprised of the cell‐tethered glycocalyx and the overlaying mucus gel; major macromolecular constituents of both components of the barrier are the O‐linked glycoproteins known as mucins. To aid site‐specific roles in the intestine, the mucus barrier is selectively organized in different regions of the GI tract and increases in thickness along its length; measurements in rats show the barrier is thickest in the colon (~830 μm) and thinnest in the jejunum (~123 μm).^12^ The small intestine has a single layer of mucus to facilitate the transition of nutrients for dietary absorption, whereas the colon has a thicker and more highly organized two‐tiered mucus barrier, composed of a firmly adherent inner layer (~50 μm) and a loose outer layer (~100 μm),^12^, ^13^, ^14^ and this organization has recently been shown to be affected by the faecal load^15^ (Figure 1). The mucus barrier architecture is required to maintain the large number of bacterial species colonizing the colon to aid symbiosis but prevent bacterial infiltration to the epithelium.^16^

Although the function of mucus has historically been accepted to act only as a physical barrier, it is now well‐recognized that it has other general intrinsic roles such as providing specific ligands for pathogen entrapment, lubrication, hydration and aiding digestion.^17^ However, exactly how the intestinal mucus barrier is organized and assembled is not fully defined, but the gel‐like properties of mucus are primarily dictated by the unique structure of the polymeric gel‐forming mucin, MUC2 (humans)/Muc2 (mice).

## 3 MUCINS

Mucins are a family of large and highly O‐glycosylated proteins that typically have a molecular weight in excess of 1 MDa. There have been 18 family members identified in humans, which have orthologues in mice and a subset of these mucins are selectively expressed at different anatomical sites along the GI tract.^18^, ^19^, ^20^, ^21^ Mucins can further be classified into 2 major subtypes: transmembrane and secreted mucins. It is the secreted mucins that form the foundation of mucus and are responsible for the characteristic rheological properties of this gel‐like secretion, while the transmembrane mucins are typically located at the apical cell surface of epithelial cells. Both subtypes of mucins contain central mucin domains, enriched in repeats of proline, serine and threonine residues (PTS or mucin domains). These domains are sites for the attachment of O‐linked glycans that results in stiffening of the protein core, which in turn leads to the enhanced space‐filling capacity of these glycoproteins which is important for their protective function.^17^

The major intestinal transmembrane mucins, MUC1, MUC3 (murine orthologue Muc17), MUC4 and MUC13, are intercalated into the apical surface of the intestinal epithelium and contribute to the glycocalyx layer.^12^, ^22^ However, the focus of this review will be the major component of the intestinal mucus gel, the secreted polymeric mucin, MUC2.^23^ It is noteworthy that MUC5AC expression can be induced within the intestine during foetal development, adenocarcinoma inflammatory bowel disease, and of specific relevance to this review, during helminth infection.^9^, ^24^, ^25^, ^26^

**Figure 2:**
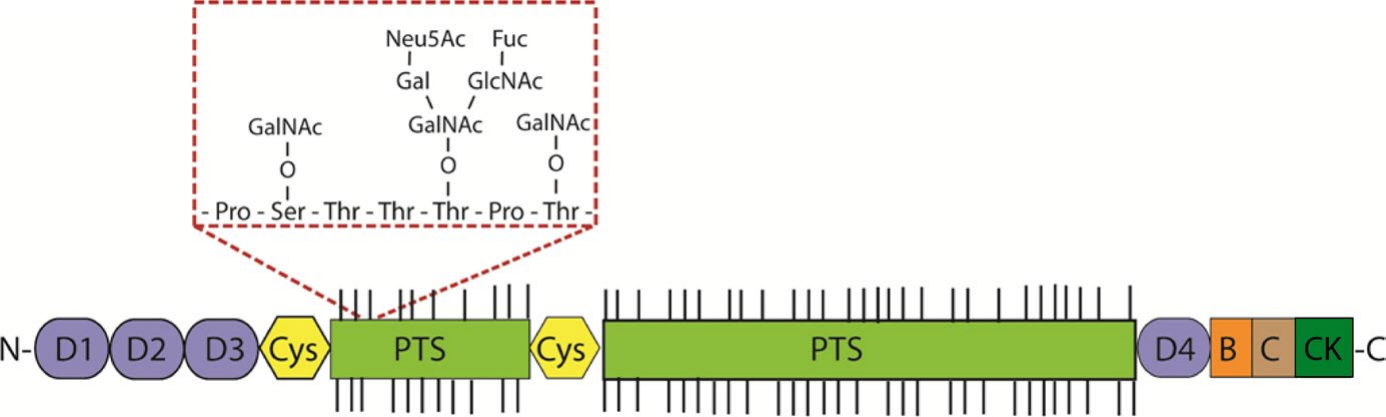
Schematic illustration of the protein domain structure of the polymeric mucin, Muc2. Muc2 consists of cysteine‐rich N‐ and C‐terminal regions. The N‐terminal region is comprised of 3 von Willebrand factor D‐domains (vWf D1‐3), and the C‐terminal domain is comprised of a vWf D‐domain (D4), a vWf B‐ and C‐domain, and a cysteine knot (CK). These terminal regions are involved in mucin polymer formation. The central heavily O‐glycosylated PTS domain is interrupted by a cysteine‐rich region (Cys‐domain). The red box highlights the glycan chains that are added onto the serine and threonine residues within the PTS domain: GalNAc (N‐acetylgalactosamine), Gal (galactose), GlcNAc (N‐acetylglucosamine), Fuc (fucose) and Neu5Ac (sialic acid)

MUC2 was the first polymeric, gel‐forming mucin to be sequenced and characterized in humans, and it shares a large degree of homology with mouse Muc2.^21^ The glycoprotein is proposed to form large, net‐like insoluble complexes, mediated by covalent linkages between mucin monomers (disulphide and isopeptide bonds).^27^ MUC2 has a well‐described domain organization, including an N‐terminal domain, 2 PTS domains (one larger than the other), 2 cysteine‐rich (Cys) domains flanking the smaller PTS domain and a C‐terminal domain (Figure 2). As mentioned previously, the PTS or mucin domains are the sites for addition of O‐linked glycans initiated through covalent attachment of N‐acetylgalactosamine (GalNAc) to either serine or threonine residues via the action of polypeptide GalNAc‐transferases (see below for further details).

The N‐terminal region of MUC2 is comprised of 3 von Willebrand factor D‐domains (vWf D1‐3), and the smaller C‐terminal region is comprised of a vWf D‐domain (D4), a vWf B‐ and C‐domain and a cysteine knot (CK). Both the N‐ and C‐terminal domains are enriched in cysteine residues that facilitate both inter‐ and intramolecular disulphide bond formation; the intermolecular disulphide linkages are responsible for mucin polymerization.^28^, ^29^, ^30^

## 4 MUC2 BIOSYNTHESIS

**Figure 3:**
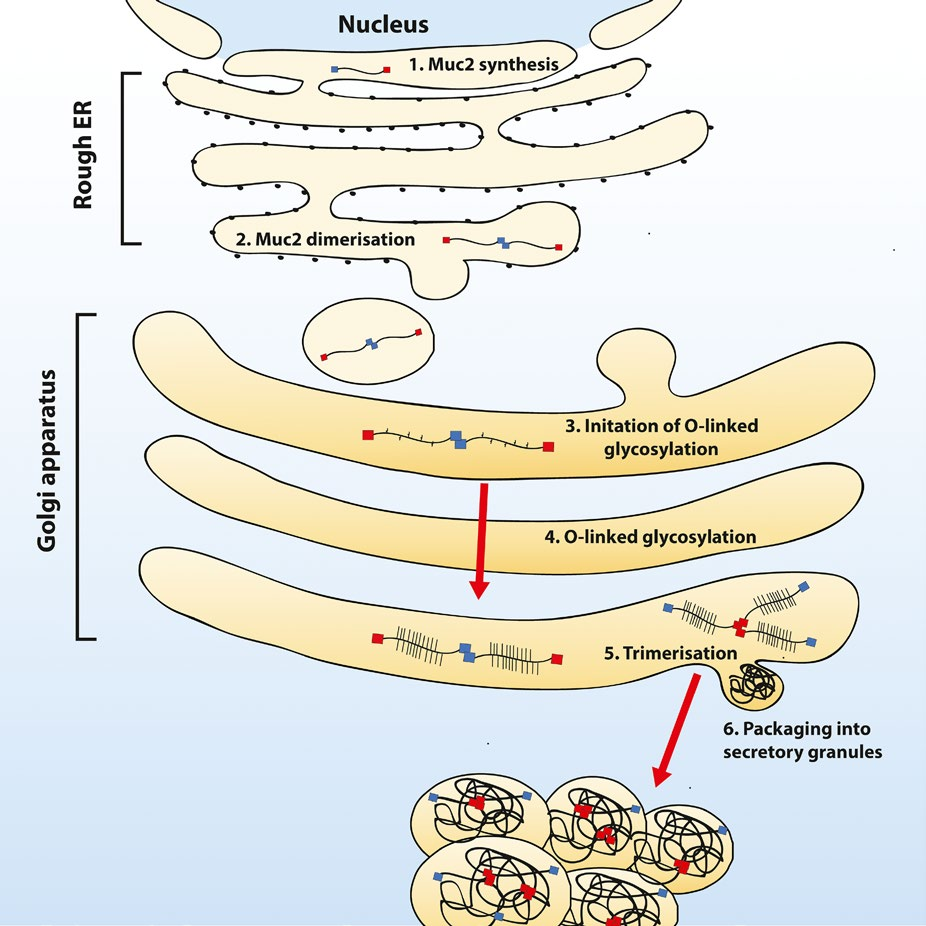
An overview of Muc2 biosynthesis in epithelial goblet cells. Monomeric Muc2 core polypeptide is synthesized in the rough endoplasmic reticulum (ER) (1), before dimerization (2) and transport into the cis‐golgi compartment where O‐linked glycosylation is initiated through the addition of GalNAc to either serine/threonine residues, (3) and extensive post‐translational modifications occur as the dimers pass through the golgi compartment. (4) At the trans‐golgi compartment, mucin dimers multimerize into large complexes (5) before becoming packaged into secretory granules prior to secretion (6)

Polymeric MUC2 undergoes a complex, multistep synthesis that puts a high‐energy demand upon the intestinal cells within which it is made and stored. As MUC2 traverses the secretory pathway, it is dimerized, extensively O‐glycosylated, further polymerized and then stored within secretory granules prior to secretion (Figure 3). Specialized cells with appropriate machinery synthesize polymeric mucins, and in the intestine, MUC2 is produced predominantly by goblet cells, which are found interspersed between enterocytes, enteroendocrine, secretory and stem cells in the intestinal epithelial layer (Figure 1); goblet cells are found at higher frequency at the most distal portions of the GI tract.

Within goblet cells, the MUC2 protein backbone is synthesized and transported to the ER, where high mannose‐type N‐linked glycosylation occurs (Figure 3, step 1). Before further modifications, MUC2 monomers will dimerize through cysteine residues located within the C‐terminal CK domain prior to transport to the golgi compartment (Figure 3, steps 2 and 3). Within the cis‐golgi compartment, the addition of GalNAc by polypeptide GalNAc‐transferases (ppGalNAc‐T) will initiate O‐linked glycosylation. Extension of the glycan backbone will occur as the protein moves through the organelle by the addition of galactose (Gal) and N‐acetylglucosamine (GlcNAc) residues, while sulphate and fucose groups can be differentially added to decorate the backbone (Figure 3, step 4). At the trans‐golgi compartment, the addition of sialic acid or GalNAc will cease glycan extension.^31^ The end result is a highly decorated protein dimer with nearly 80% of the mass accounted for by glycans.^32^ These glycan chains are highly heterogeneous in chain length and composition even at homeostasis. Importantly, glycan structure can be influenced during parasitic infections, which aids host protection against pathogenesis (which will be discussed further below).^33^, ^34^, ^35^, ^36^

Finally, MUC2 dimers have been proposed to trimerize through disulphide bonds mediated by cysteine residues located in the N‐terminal vWfD3 domain and isopeptide bonds formed between the side chains of lysine and glutamine residues (Figure 3, step 5).^27^ These covalent linkages give rise to very large and highly glycosylated polymers,^37^, ^38^ which are packaged in dehydrated form inside secretory granules (Figure 3, step 6). This storage mechanism allows for the release of fully synthesized MUC2 polymers which undergo rapid hydration and expansion on the intestinal epithelial surface to maintain mucus barrier integrity during homeostasis or barrier breach.^39^ Hydration and expansion of MUC2 polymers is dictated by the ionic composition and water availability at the intestinal epithelial surface, and after secretion, mucin polymers can expand their volume up to 1000 times, becoming entangled within one another to form the structural framework of the mucus gel.^39^, ^40^

The control of mucin secretion has yet to be fully defined, but it is evident that intestinal goblet cells secrete MUC2 at a basal rate during homeostasis, but the major route during environmental and infectious challenge is by regulated secretion via compound exocytosis.^41^ The secretion of MUC2 can be influenced by a broad range of mediators including cytokine signals, microbial‐derived products, adrenocorticotropic hormones, autophagic proteins, reactive oxygen species and components of the inflammasome (NOD‐, LRR‐ and pyrin domain‐containing 6).^10^, ^11^, ^42^, ^43^, ^44^, ^45^, ^46^

Recent research in mice suggests that there may be different types of intestinal Muc2‐secreting goblet cells and their function and secretory activity are dictated by their location within the intestinal crypt.^47^ Birchenough and colleagues describe “sentinel” goblet cells that are located at the top of the colonic crypts and secrete Muc2 after bacterial‐induced activation of Nlrp6 inflammasome via TLR/MyD88 signalling axis.^47^ It has yet to be determined whether these different types of goblet cell have different gene signatures, or may arise due to the natural maturation of the goblet cell as it moves up the crypt during epithelial cell turnover. Further research using intestinal‐derived 3‐D enteroid cultures will provide a simple and manipulable system to directly assess signalling cues that are required for goblet cell function and help our understanding of goblet cell biology during inflammatory threat.^48^

## 5 IMMUNE CONTROL AGAINST GI NEMATODE INFECTION

Infection with GI nematodes is commonly associated with the generation of type 2 immunity; the cytokine IL‐13 is a critical driver for this response and is primarily derived from type 2 innate lymphoid cells (ILC2) and Th2 cells. The induction of IL‐13 and IL‐4 leads to the expansion of goblet cells, a trait that has been observed during *N. brasiliensis*,* T. spiralis* and acute *T. muris* infections.^10^, ^49^, ^50^ As goblet cells are the major mucin‐producing cells in the intestine, this expansion leads to alterations in mucus barrier properties, through secretion of mucins and other goblet cell‐associated proteins. In recent years, there has been significant progress in understanding the initial mediators of goblet cell hyperplasia during GI nematode infection.

**Figure 4:**
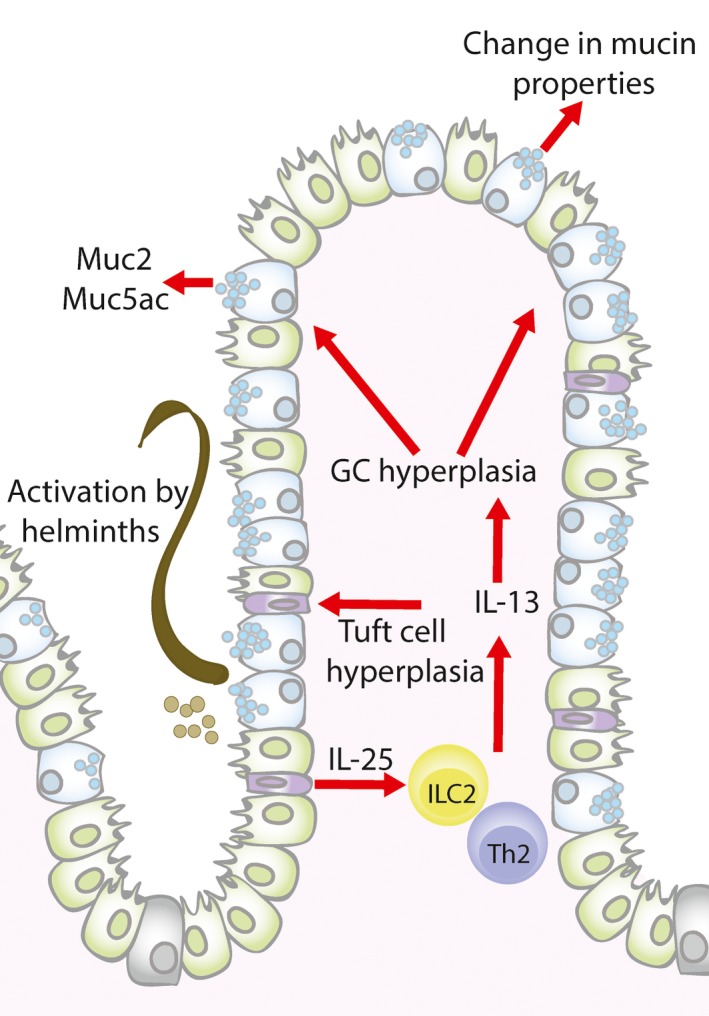
Mucosal alterations during intestinal helminth infections. After helminth infection tuft cells will secrete IL‐25, which leads to the production of type 2 cytokines, predominately ILC2 derived IL‐13, which together with Th2 derived IL‐13 leads to goblet cell (GC) and tuft cell hyperplasia and changes in mucin production (Muc2 and Muc5ac) and mucin properties (glycosylation). These alterations change the properties of the mucus barrier to aid host protection against intestinal nematode infection

ILC2s were originally identified as an alternative source of type 2 cytokines in mice lacking T or B cells^51^ and have been demonstrated to be a critical source of IL‐13 and IL‐13‐driven goblet cell responses during GI nematode infections. ILC2s can be primed and activated after stimulation with epithelial‐derived cues; IL‐25, IL‐33, TSLP.^52^ More recently, neuropeptide neuromedin U signalling has been indicated to be a potent type 2 cytokine initiator; capable of causing activation and proliferation of ILC2s, and associated with accelerated expulsion of *N. brasiliensis*.^53^ ILC2s are primed early during *H. polygyrus* 54 and *N. brasiliensis* infection,^55^ providing a source of IL‐13 which promotes the production of type 2 cytokines and goblet cell hyperplasia. More recently, data has emerged to suggest tuft cells are critical in orchestrating signalling cues for type 2‐mediated immunity during GI nematode infections, facilitating the communication between the epithelium and the underlying immune cells.^56^ Tuft cells are a chemosensory cell of the gut,^57^ and the induction of tuft cells has been demonstrated to provide an early supply of IL‐25 during *N. brasiliensis*,* T. spiralis* and *H. polygyrus* infection, which in turn leads to the induction of IL‐13 producing ILC2s and results in a feedforward system to cause tuft cell hyperplasia^54^, ^55^ (Figure 4). Furthermore, goblet cell hyperplasia during *N. brasiliensis* infection is dependent on the presence of tuft cells, as mice deficient in tuft cells (Pou2f3^−/−^ mice) not only have reduced IL‐25 expression and ILC2 expansion, but goblet cell hyperplasia did not occur and animals were unable to expel the parasite, unlike their wild‐type counterparts.^58^ Interestingly, single‐cell RNA‐sequencing data have revealed that there are 2 different subtypes of tuft cells, with tuft‐2 cells being significantly upregulated during *H. polygyrus* infection and shown to express the epithelial cytokine Tslp and the pan‐immune marker CD45 (an atypical nonhematopoietic cell marker),^59^ which highlights an additional level of complexity of this cell type and its role in immunity.

It is important to note that although it is widely accepted that the IL‐13/IL‐4 signalling axis is the primary mediator of goblet cell hyperplasia, and therefore effector functions,^49^, ^60^, ^61^ there is evidence to suggest that other immune mediators can coordinate goblet cell effector functions. For example, a study conducted by Marillier and colleagues demonstrated goblet cell hyperplasia is observed independent of the IL‐13/IL‐4 signalling apparatus,^62^ and *Muc2* and *Muc3* transcripts were augmented even in the absence of IL‐4 during *T. spiralis* infection.^50^ Furthermore, inflammatory cytokines have been implicated to have a role in goblet cell function during parasitic infection, including IL‐1, TNF and IL‐22,^63^, ^64^, ^65^ and it is yet to be defined if microbial factors, the inflammasome and adrenergic and cholinergic receptors can influence the secretion of mucins and other goblet cell‐associated products during parasitic invasion.

## 6 GI NEMATODE INFECTION AND MUCUS

Despite the long‐standing mucus‐trap hypothesis, there are still many gaps in our knowledge of the precise functional role(s) of the mucus barrier during host protection against GI nematodes. Studies have shown that IL‐13 increased mucus production during *N. brasiliensis* expulsion^60^, ^66^ and this mucus was hypothesized to interfere with the parasites ability to associate with the intestinal epithelium and feed.^67^ Furthermore, the mucus barrier has been overserved to interfere with invasive stages of *T. spiralis* infection, and *T. muris* infection has been shown to alter the porosity of the mucus barrier but only at the site of parasite colonization^35^; in acute *T. muris* infection, there was more caecal mucus and the mucus barrier was less porous compared to chronic infection.

Although the primary role of the mucus barrier is to act as a physical barrier to protect the underlying intestinal epithelium, it has also been demonstrated that the mucus barrier can actively lead to deleterious effects on parasite viability. For example, rat intestinal mucus was detected within the intestine of *N. brasiliensis* worms and linked to morphological damage to the adult worm gut cells during the development of host immunity.^68^, ^69^ Furthermore, inflammatory mediators held within the mucus barrier have been demonstrated to have paralysing activity against nematodes.^70^ These findings suggest that mucus is a crucial innate defence mechanism against invading GI parasitic nematodes and the known roles of different components of the mucus barrier are discussed below.

## 7 GI NEMATODE INFECTION AND MUCINS

As mucins are the structural framework of the mucus barrier and the epithelial cell glycocalyx, multiple studies have examined the role of these glycoproteins during GI parasite infection. For example, *Muc2* and *Muc17* transcripts are significantly upregulated during *T. spiralis* infection in mice,^50^ and in an experimental *T. spiralis* infection in pigs, there was an increase in goblet cell‐stored mucins and a change in the glycosylation pattern of the mucin glycans within the small intestine.^71^ Moreover, using a porcine model of *Trichuris* infection, *Trichuris suis*, there was a significant upregulation of mucin production,^72^ and in mice, *T. muris* infection caused an increase in the levels of *Muc2* transcripts only at the site of the parasite colonization (ie the mouse caecum) at the time of worm expulsion. Perhaps surprisingly, ablation of Muc2 in vivo only led to a delayed *T. muris* expulsion during acute infection, even though a Th2‐mediated immune response prevailed.^73^ Further studies showed there was an induction of the polymeric mucin Muc5ac, normally a gastric and lung mucin, which was important for *T. muris* parasite expulsion and suggested a protective function for this mucin in the intestine.^9^ This was confirmed using Muc5ac‐deficient mice, which were completely susceptible to *T. muris* infection unlike their wild‐type counterparts. Importantly, the susceptible phenotype was not reversed even after administration of anti‐IFN‐γ to skew the Th1 dominated environment generated in chronic infection towards a Th2‐directed immune response, normally associated with resistance.^10^ Importantly, there was an induction of *Muc5ac* transcripts in *T. suis* infected pigs, suggesting a protective role across species,^74^ and there is data to suggest that Muc5ac may also have a broad antihelminth action as Muc5ac null mice were also impaired in their ability to efficiently expel *N. brasiliensis* and *T. spiralis*.^9^

The invading GI nematodes are likely, therefore, to employ strategies to subvert the mucus barrier to allow them to establish within their intestinal niche, which facilitates the complex interplay between host and parasite. For example, in chronic *T. muris* infection the parasite secretes excretory/secretory products (E/S) that contain proteases, such as serine proteases. These proteases can degrade the polymeric Muc2 network and hence increase the porosity of the barrier aiding establishment of the parasite in the caecal epithelium. During acute infection, however, Muc5ac is not degraded by *T. muris* E/S derived proteases. In addition, the resistant mice showed a significant upregulation of serine protease inhibitors (ie serpins) that protect mucin polymers from degradation maintaining the integrity of the mucus barrier.^35^ Further work is required to elucidate the signalling cues employed to initiate Muc5ac expression within the intestine, as this pathway could be a potential therapeutic target to induce parasite expulsion in humans and domestic animals.

## 8 GI NEMATODE INFECTION AND ALTERATIONS IN MUCIN GLYCOME

A unique feature of mucins is the heterogeneous array of glycan structures that decorate the polypeptide backbone, and these glycans have well‐established roles in influencing pathogenic organisms, including GI nematodes.^10^, ^75^ Mucin glycans have been highlighted to be significantly altered during multiple inflammatory responses within the intestine, including during infection with *N*. *brasiliensis*,* T*. *spiralis*,* T. muris* and *H*. *polygyrus*.^76^, ^77^, ^78^, ^79^ It has yet to be determined whether these changes occur as a result of the inflammatory environment or to resolve the infection; clearly more research is required to define the precise role of mucin glycans. Most of our current understanding of the central role that mucin glycosylation plays during inflammatory threat has come from challenging rodents with infectious agents,^71^, ^77^, ^78^ and there are data to suggest that during GI nematode infections, there are multiple changes in the expression of glycosyltransferases, the enzymes responsible for the synthesis of the glycan chains.^75^ Yamauchi et al^77^ have demonstrated that by day 2‐3 of *T. spiralis* infection, there is an increase in α‐2‐3‐sialyltransferase IV. Additionally, during maturation of *T. spiralis,* there is also an induction of 3‐0 sulphotransferase‐1 expression in the intestine that peaks at day 14 post‐infection and falls only when parasite expulsion occurs.^77^ Furthermore, *Dolichos biflorus* agglutinin (DBA) and Muc2 dual staining showed a higher prevalence of GalNAc residues on Muc2 in acute *T. muris* infection compared to chronic infection.^10^ The functional consequences of these changes remain to be elucidated.

One aspect of mucin glycosylation that has received most attention from researchers during GI nematode infection is the sialic acid and sulphate content of the Muc2 O‐glycans. The relative ratio of these negatively charged species has been hypothesized to influence parasite establishment. An in vitro study has demonstrated that a decrease in the level of sulphated mucins (sulphomucins) leads to a reduction in the establishment of *Strongyloides venezuelensis,* 80 with the degree of sulphation affecting the time of parasite expulsion in Syrian golden hamsters.^81^ Furthermore, sulphotransferases are induced prior to *N. brasiliensis* expulsion,^82^, ^83^ and the induction of specific sialic acid‐containing mucins (sialomucins) bearing Sd^a^ blood group antigens, driven via the IL‐13/4 receptor axis, has also been correlated with parasite expulsion.^84^, ^85^ During chronic *T. muris* infection, there is a change from sulpho‐ to sialomucins restricted to the niche of the parasite. In contrast, during acute *T. muris* infections there is maintenance of the level of sulphomucins, driven by IL‐13. Importantly, Muc2 containing the sulphated glycans is less susceptible to parasite‐mediated proteolytic degradation than its sialomucin counterpart that dominates in chronic infection.^75^ Furthermore, in a murine model with reduced mucin sulphation at homeostasis due to a genetic deletion of sulphate anion transporter 1 (Sat‐1), mice that would normally be resistant to infection become susceptible, despite the prevailing Th2 immune response.^75^ These changes in mucin glycosylation lead to global changes in the mucosal barrier, affecting mucin charge density, which leads to direct alterations within the barrier, including mucus hydration and viscosity that in turn may hinder the parasites’ ability to degrade the mucus and thus contribute to host protection.

## 9 GI NEMATODE INFECTION AND MUCUS‐ASSOCIATED PROTEINS

Although the major structural component of the intestinal mucus gel is MUC2/Muc2, the viscous gel is a multifaceted mixture of molecules that contains water, electrolytes, carbohydrates, proteins, nucleic acids, amino acids and lipids.^17^ Proteomic analyses of mucus samples derived from the large intestine have demonstrated that there are hundreds of proteins held within the mucus gel during homeostasis.^86^ However, it is important to note that as the intestinal tract is such a dynamic and exposed tissue, it is also likely that the mucus gel will contain exfoliated cells from the rapidly turned over epithelial layer, bacterial‐derived products and dietary components, making it difficult to extrapolate which proteins are functionally important. However, proteins with a structural, antimicrobial and regulatory function have been associated with the mucus gel and have been identified to be present during parasitic infection.^87^, ^88^, ^89^, ^90^, ^91^ Several nonmucin proteins have also been demonstrated to be present within goblet cell granules within the intestine, including IgG Fc‐gamma‐binding protein (FCGBP), trefoil factor (TTF), chloride channel regulator calcium‐activated‐1 (CLCA1), resistin‐like molecule (RELM)‐β and ZG16,^87^, ^92^, ^93^, ^94^ but relatively few have been investigated during GI nematode infection.

RELM‐β is found within ceacal and colonic mucus as a hexamer and trimer and is induced by a Th2 response.^94^ It has been proposed that RELM‐β can affect the ATP levels and hence the fitness of *H. polygyrus* and *N. brasiliensis* through impairing the parasites ability to feed. Moreover, RELM‐β can aid host protection against *N. brasiliensis* by causing entrapment of the parasite and reducing parasite motility.^88^ However, during *T. muris* and *T. spiralis* infection there is an induction of RELM‐β expression, but it appears to play little role in expulsion.^87^ Additionally, the expression of antimicrobial agents derived from goblet cells, namely angiogenin 4, intelectin‐1 and intelectin‐3, have been associated with *T. muris* expulsion, but their functional importance has yet to be determined.^89^, ^90^, ^95^

Trefoil factors (TFFs) are a family of 3 cysteine‐rich proteins, TFF1, TFF2 and TFF3, having roles in mucosal repair and protection against GI insult.^96^ Studies have demonstrated that TFFs interact with mucins to aid mucus gel integrity.^92^ For example, TFF3 has been demonstrated to promote mucosal barrier protection during a rat colitis model.^97^ Despite this, *T. spiralis*‐infected TFF3 knockout mice had no clear phenotype in comparison with their wild‐type counterpart during infection.^91^ It has been suggested, however, that TFF2 plays important roles during the lung stage of *N. brasiliensis* infection, which has been correlated with augmenting both IL‐33 and Muc5ac expressions within the lung.^98^

## 10 CONCLUSION

Taken together, these data suggest that goblet cells and their secreted products, in particular the polymeric mucins, are important elements for initial protection against GI helminths, and necessary for subsequent clearance of parasites during infection. Not only is there a change in mucus barrier composition and properties, but there is also changes in mucin expression and glycosylation during GI nematode infections. These changes in the mucus barrier constitute a coordinated and critical arm of the innate immune effector response against GI helminths. A better understanding of the regulatory pathways involved in eliciting these changes could highlight novel therapeutic targets to help eradicate this prevalent group of parasites.

## DISCLOSURES

The authors declare no conflict of interest.
